# Bovine Respiratory Syncytial Virus (BRSV) Infection Detected in Exhaled Breath Condensate of Dairy Calves by Near-Infrared Aquaphotomics

**DOI:** 10.3390/molecules27020549

**Published:** 2022-01-16

**Authors:** Mariana Santos-Rivera, Amelia R. Woolums, Merrilee Thoresen, Florencia Meyer, Carrie K. Vance

**Affiliations:** 1 Department of Biochemistry, Molecular Biology, Entomology, and Plant Pathology, Mississippi State University, Starkville, MS 39762, USA; jms2033@msstate.edu (M.S.-R.); fsm28@msstate.edu (F.M.); 2 College of Veterinary Medicine, Pathobiology & Population Medicine, Mississippi State University, Starkville, MS 39762, USA; aw1873@msstate.edu (A.R.W.); mt1657@msstate.edu (M.T.)

**Keywords:** absorbance, biofluid, cattle, chemometrics, discrimination, NIRS, transmittance, virus

## Abstract

Bovine respiratory syncytial virus (BRSV) is a major contributor to respiratory disease in cattle worldwide. Traditionally, BRSV infection is detected based on non-specific clinical signs, followed by reverse transcriptase-polymerase chain reaction (RT-PCR), the results of which can take days to obtain. Near-infrared aquaphotomics evaluation based on biochemical information from biofluids has the potential to support the rapid identification of BRSV infection in the field. This study evaluated NIR spectra (*n* = 240) of exhaled breath condensate (EBC) from dairy calves (*n* = 5) undergoing a controlled infection with BRSV. Changes in the organization of the aqueous phase of EBC during the baseline (pre-infection) and infected (post-infection and clinically abnormal) stages were found in the WAMACS (water matrix coordinates) C1, C5, C9, and C11, likely associated with volatile and non-volatile compounds in EBC. The discrimination of these chemical profiles by PCA-LDA models differentiated samples collected during the baseline and infected stages with an accuracy, sensitivity, and specificity >93% in both the calibration and validation. Thus, biochemical changes occurring during BRSV infection can be detected and evaluated with NIR-aquaphotomics in EBC. These findings form the foundation for developing an innovative, non-invasive, and in-field diagnostic tool to identify BRSV infection in cattle.

## 1. Introduction

Bovine respiratory syncytial virus (BRSV) is an enveloped, non-segmented, negative-stranded RNA virus that belongs to the order *Mononegavirales*. It is a member of the *pneumovirus* genus within the *Pneumovirinae* subfamily of the *Paramyxoviridae* family [1,2]. The BRSV virion is made up of a lipid envelope generated from the host plasma membrane; it contains three virally encoded transmembrane surface glycoproteins that are arranged independently on the surface as spikes. These glycoproteins are the large glycoprotein G, the fusion protein F, and the small hydrophobic protein SH [1,2]. BRSV is very contagious and spreads through respiratory aerosols and via animal-to-animal contact [3]. During the incubation period (2–5 days), BRSV replicates primarily in ciliated respiratory epithelial cells and type II pneumocytes. After incubation, BRSV infection can be asymptomatic, or clinical signs such as fever (39.4–42.2 °C) and cough with a seromucoid nasal and ocular discharge can be present [2]. Clinical signs are typically mild; however, immunological suppression and mucosal damage in the respiratory tract might lead to subsequent bacterial infection, leading to BRDC (bovine respiratory disease complex) [1,4]. BRDC is the most costly disease affecting young calves worldwide [5,6,7]. As a result, it is critical to design new ways to prevent the spread of the primary causes of infection by this complex, which requires fast and non-invasive diagnostic tools to detect early cases of BRSV infection in the herd.

Field diagnosis of BRDC and BRSV infection is conventionally based on visual-clinical diagnosis (VCD) of the appearance and behavior of cattle, which is not specific for detecting and treating the causal agent [8,9,10]. Specialized techniques to detect BRSV infection have been developed and may include lung lavage, tracheal washes, and nasal swabs collected from live cattle or post-mortem samples for virus isolation and recognition of cytopathic effects in cell cultures [1,3]. Serological tests such as immunofluorescent antibody (IF) and enzyme-linked immunosorbent (ELISA) assays are also used to detect viral infection, exhibiting 47% and 60% sensitivity for BRSV, respectively. By comparison, analysis using reverse transcription-polymerase chain reaction (RT-PCR), which is currently considered the gold standard for BRSV diagnosis, has 99% sensitivity and specificity [1,3,11]. More recently, analytical techniques such as nuclear magnetic resonance (NMR) and gas chromatography based mass spectrometry (GC-MS) have been used in blood plasma, blood serum, nasal secretions, and exhaled breath condensate to identify biomarkers related to BRDC to create new diagnostic tools that facilitate appropriate treatment [12,13,14,15,16,17].

Near-infrared spectroscopy (NIRS) is a type of vibrational spectroscopy that measures absorption resulting from the interaction of NIR light (700–2500 nm) with functional groups of organic matter [18,19]. The application of NIRS to biological systems has often been confounded by the strong overtone signal of the water matrix, which acts to not only solvate biomolecules through hydrogen bonding but also has a complex and dynamic microstructure [18]. Aquaphotomics is a recent discipline in which the water spectrum arises from the interactions of water with specific biological components, while also focusing on how the biological composition influences the formation of water coordination spheres, clusters, and ions, which have distinct absorbance bands described by the water matrix coordinates (WAMACS) [20,21,22,23]. Thus, aquaphotomics is being increasingly applied to detect changes in water-based systems and is particularly applicable to biological fluid matrices in which a suite of biochemical changes occurs in response to shifts in organismal homeostasis [24,25].

Exhaled breath condensate (EBC) can be collected non-invasively and has been used to monitor respiratory disease in humans and animals [26,27,28,29]. EBC contains metabolites or substances such as adenosine, ammonia, cytokines (small proteins ~5–25 kDa), hydrogen peroxide, isoprostanes (free radical lipid peroxidation of arachidonate), leukotriene B4, nitrogen oxides (NO_2_^−^ and NO_3_^−^), and peptides demonstrated to be helpful in evaluating oxidative stress and inflammatory response in the respiratory system [26,30], and which have been detected by GC-MS [14,16,31,32,33]. We propose that with these known indicators of inflammation evident in EBC, NIRS-based aquaphotomics coupled with chemometrics may provide a mode of rapid analysis of respiratory disease that can be conducted in the field. In this research, dairy calves were challenged with BRSV, and the EBC samples that were collected before and after infection were assessed by NIRS-aquaphotomics to create disease profiles and prediction equations, with a specific inquiry into the early stages of disease progression.

## 2. Materials and Methods

### 2.1. Animals and Controlled Challenge

**Figure 1:**
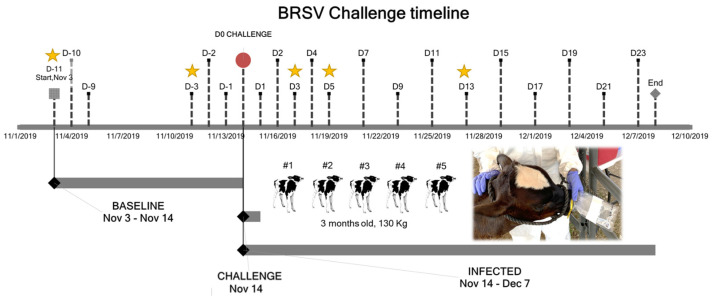
Timeline for the BRSV controlled challenge carried out in five dairy calves. The stars point out the days of the EBC collection.

BRSV strain GA-1, P5 was propagated in Madin-Darby bovine kidney cell lines grown in Dulbecco’s minimal essential medium with 10% fetal bovine serum and 2 mM of Gibco™ GlutaMAX™ Supplement. On day 7 post-inoculation of cells, the cell culture supernatant containing −5.20 TCID_50_ units of BRSV per mL was collected. Then, 5 mL of the supernatant was administered via a nebulizer (DeVilbiss Pulmo-Neb) through a custom-made face mask to each of five non-vaccinated three-month-old Holstein steers weighing approximately 130 kg and housed in outdoor pens isolated from other cattle at Mississippi State University (Animal Care and Use Committee IACUC-19-037) [17]. Clinical signs (VCD) of respiratory infection, including rectal temperature, heart rate, respiratory rate, assessment of nasal and ocular discharge, presence of cough, breathing pattern, and character of lung sounds, were evaluated before and after infection (Figure 1) [17,34].

### 2.2. Exhaled Breath Condensate (EBC) Collection

Samples were collected before and after the controlled challenge (D0). For the collection of EBC (*n* = 24), a sterile Nasco Whirl-PaK^®^ sample bag with puncture-proof tabs (cat# B01489) was placed over one of the calf’s nostrils for 15 min, ensuring that the breath was blown into the collection bag (Figure 1). The bag with the aerosolized particles was stored at −80 °C for 24 h, then thawed at room temperature (21 °C) to allow the condensation of the particles forming liquid droplets. The droplets (approximately 500 µL total volume) were stored in sterile Eppendorf tubes at −80 °C until NIRS analysis. To avoid discomfort in the dairy calves, these samples were collected only five times on the following days: D-11, D-3, D3, D5, and D13.

### 2.3. Spectral Signature Acquisition

Transmittance NIR spectra (*n* = 240) were collected from 300 µL of EBC using a portable ASD FieldSpec^®^3 spectrophotometer and Indico^®^Pro software (Malvern Panalytical, ASD Analytical Spectral Devices Inc. Boulder, CO. USA) as previously described by the authors [32]. Ten spectral signatures were collected independently per sample. Each NIR spectrum was taken across the wavelength range 350–2500 nm (interval = 1.4 nm for the region 350–1000 nm and 2 nm for the region 1000–2500 nm; 50 scans; 34 ms integration). Spectral signatures from sterile distilled water were collected as the reference solution required for the aquaphotomics analysis [34].

### 2.4. Data Analysis

Based on the clinical signs (VCD) evaluated before and after the BRSV challenge, the samples were classified as having been collected during the baseline, Asymptomatic, infected, or Recovered stages relative to challenge. To avoid interference of the asymptomatic phase or the recovery process in the interpretation of the aquaphotomics profiles, only an equal number of samples designated as baseline (pre-infection) and infected (post-infection and clinically abnormal) were used in the aquaphotomics evaluation and the statistical and multivariate analyses (MVA) [34]. The absorbance of EBC and the reference solution was transformed with the mathematical pre-treatments of SNV (Standard Normal Variate) with de-trending (polynomial order: 2), baseline Offset & Linear baseline Correction, and a 2nd derivative (polynomial order: 2, Gap Size: 25, Segment Size: 19, Savitzky-Golay smoothing points: 25) (Unscrambler^®^ X v.11, Aspen Technology Inc, MA, USA).

### 2.5. Aquaphotomics Approach

Aquaphotomics was applied to determine the molecular organization of the aqueous phase of EBC as a method to identify the biochemical changes caused by the infection. PCA (principal component analysis) was performed on the mean-centered matrix of the aquaphotomics region (1300–1600 nm) using full random cross-validation and the algorithm SVD (singular value decomposition) as a first step in revealing trends in the spectra with the goal of exploring the data, identifying dominant peaks in the loadings, and looking for outliers (Hotelling’s T^2^ influence plot) [23]. Previously described WAMACS were used to create barcodes and aquagrams using a spectral database containing samples from all the calves (*n* = 200) [20,21,23,34]. Using Microsoft Excel 365^®^, the absorbance was normalized by subtracting the mean transformed absorbance of distilled sterile water as the reference solution from the mean transformed absorbance of each group (baseline or infected) and then dividing the results by the standard deviation (SD) of each category [21,23]. For the barcodes, WABS (water absorbance bands) within the WAMACS were identified by plotting the normalized absorbance to identify the direction of the peaks before detecting the WABS in each point of absorbance of the 12 WAMACS. These identified points were colored in the barcode according to each category to compare chemical shifts [34]. For the aquagram, the identified WABS for the baseline were selected as the points to be plotted in a radar chart to obtain the WASPS (water absorbance spectral patterns), which indicate the trends in the organization of the water molecules of the evaluated samples [21,23].

### 2.6. Chemometrics

The chemometric analysis was performed on the first overtone region of the near-infrared transformed absorbance between 1300 and 1600 nm. Balanced databases (*n* = 200) were generated to control the disproportion and diversity of the total number of samples collected [34]. These databases were used to create five datasets by stratified random sampling and analyzed in a leave-one-animal-out approach to develop five PCA-LDA (linear discriminant analysis) models using the Mahalanobis method [35,36]. To produce the calibration and internal validation sets (128/32), spectra from four calves were partitioned into an 80/20 percent distribution; spectra from the fifth calf were utilized as the external validation set (*n* = 40) (Unscrambler^®^ X v.11.0, Aspen Technology Inc, Bedford, MA, USA). Within each model, nine predictions were tested using PCs (principal components), explaining from 95% to 99.9% of the variation of the databases using the top-down approach for PC selection methodology [34,37]. A total of 45 predictions were evaluated; however, only the results from the ones with the best performance are shown in the results section. Quality parameters, including the percentage of accuracy, sensitivity, and specificity, were carried out to establish the technique’s ability to classify true positive and true negative samples [34,38]. ANOVA, and a pairwise mean comparison using Tukey-Kramer HSD (honestly significant difference) test with alpha = 0.05 were used to assess significance between models (JMP^®^ 14.0, SAS Institute Inc., Cary, NC, USA); in addition, a pairwise mean comparison using Student’s t-test with alpha = 0.05 was used to evaluate differences between categories (baseline vs. infected).

## 3. Results

### 3.1. Aquaphotomics Findings

**Figure 2:**
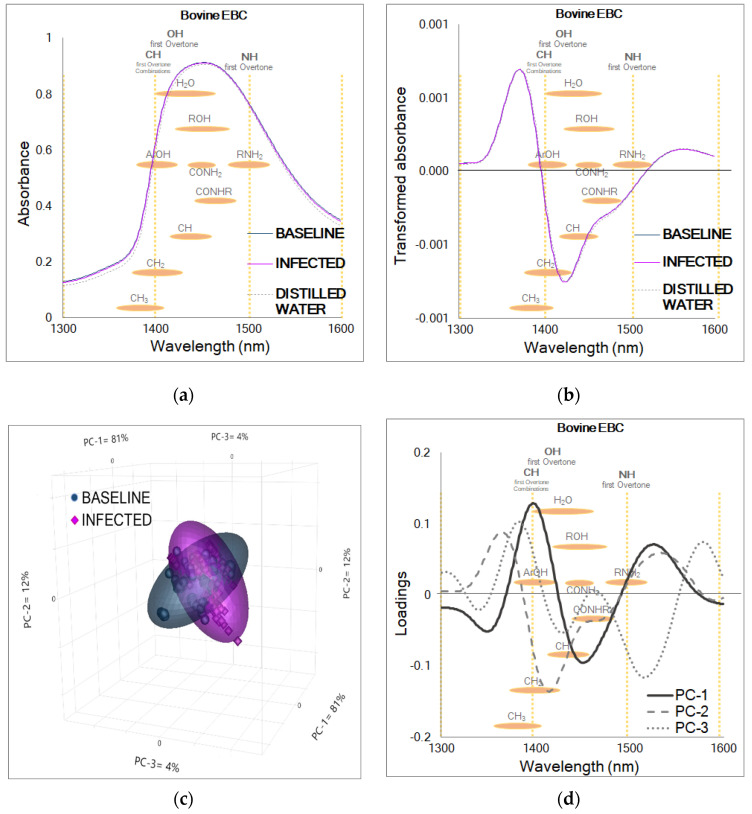
NIR absorbance from bovine EBC collected before and after BRSV infection. (**a**) Raw or unprocessed NIR absorbance from baseline (*n* = 100) and infected (*n* = 100) stages relative to infection, showing the characteristic water spectral pattern. (**b**) Transformed or processed absorbance from baseline (*n* = 100) and infected (*n* = 100) stages displaying two prominent features at 1376 and 1424 nm. (**c**) PCA scores plot for samples of the baseline and infected stages from all the calves (*n* = 5) challenged in this study (*n* = 200). (**d**) PCA loadings showing the dominant peaks influencing the positive and negative trends in the scores plot: PC-1 = 81%, PC-2 = 12%, PC-3 = 4%.

The aquaphotomic analysis for NIR spectral signatures collected for breath condensate from dairy calves infected with BRSV revealed a consistent and expected spectral water pattern in the wavelength range between 1300 and 1600 nm (Figure 2a). This region corresponds to the first overtone of the functional groups O-H, C-H, and N-H forming molecules containing water (H_2_O), alcohols (ROH), phenols (ArOH), simple amides (CONH_2_), amides (CONHR), monoamides (RNH_2_), methylene (CH_2_), and methyl radicals (CH_3_) [18], which are, in this case, likely related to the high content of water and the volatile and non-volatile compounds in the EBC [30]. The pattern in the transformed absorbance (Figure 2b) was also expected due to the application of a second derivative and Savitzky-Golay smoothing in the pre-treatment [39]. Chemical similarities were observed in the trends of the PCA scores plot (Figure 2c), where both categories overlap. The dominant peaks identified in the positive and negative directions of the PCA loadings (PC-1 = 1347, 1396, 1454, 1528 nm; PC-2 = 1396, 1418, 1539 nm; PC-3 = 1338, 1380, 1431, 1524, 1576 nm) explained the aforementioned patterns, with the first three PCs accounting for 97% of the variation in the spectral database (Figure 2d). There were no outliers in the Hotelling’s T^2^ influence plot (not shown). These findings highlight the need to use both, aquaphotomics and chemometrics-based MVA to reveal discriminating biochemical profiles of BRSV infection in EBC.

**Figure 3:**
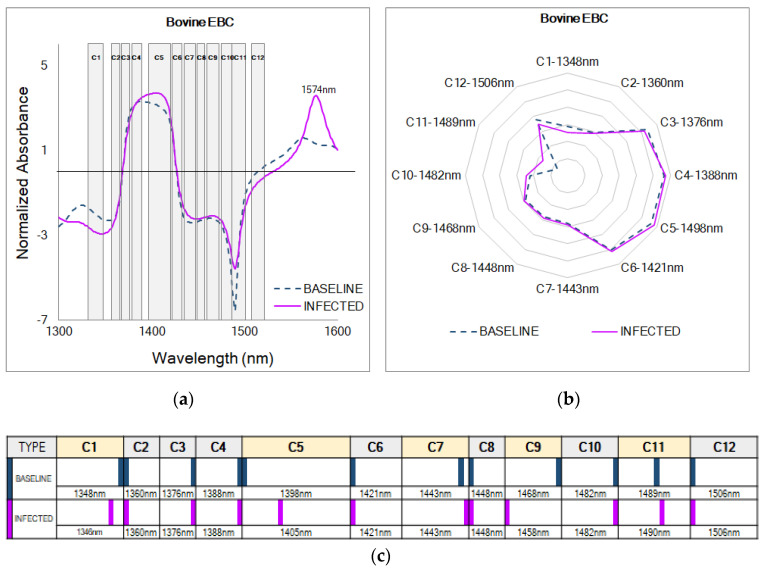
Aquaphotomics of EBC from dairy calves infected with BRSV. (**a**) Normalized absorbance for samples from the baseline (*n* = 100) and infected (*n* = 100) stages from all the calves (*n* = 5) challenged in this study. (**b**) Aquagram created with the key WABS from the baseline stage. (**c**) WAMACS barcode showing chemical shifts.

The normalized absorbance of EBC collected in the pre-infection baseline stage and the infected stage when calves had clinical signs of disease revealed different patterns for both categories, indicating differences in the chemical composition of EBC from healthy and sick dairy calves (Figure 3a). Interestingly, at 1574 nm, a prominent feature in the normalized absorbance from the infected stage was unveiled; this wavelength corresponds to the first overtone of N-H stretching vibration and is not included in the current described WAMACS.

The WASPS in the aquagram (Figure 3b) indicate that water molecules in EBC from both categories were highly organized in the WAMACS C3, C4, C5, and C6 and less organized in the coordinates C7 to C12, which are related to water dimers (S_1_) and water clusters with two, three, and four hydrogen bonds (S_2_, S_3_, and S_4_) [21,23]. In this case, baseline samples showed the highest absorbance at C4, and samples from the infected stage showed the highest absorbance at C5. At C4 (1380–1388 nm), a change in the formation of complex three-dimensional molecular spheres or hydration shells around solute molecules can be implied, mainly concerning the lipid (total lipids, phospholipids, cholesterol, triglycerides, eicosanoids, free fatty acids) and cytoplasmic (glucose, ammonia, lactate; pyruvic, succinic, and oxaloacetic acids) metabolites known to increase in EBC during chronic pulmonary disease [21,40,41,42,43,44]. At C5 (1398–1418 nm), two types of changes in the water matrix of EBC can be inferred, the first one related to water molecules confined in a local field of ions and the second related to water molecules with free hydroxide ion (OH-) [21,23]. Moreover, higher absorbances at C3 (1370–1376 nm) suggest that molecules were structured in water symmetrical and asymmetrical stretching vibrations (ν_1_ + ν_3_) related to variations in the EBC solute composition during the baseline or infected stage [21,23]. In addition, at C6 (1421–1430 nm), changes in the HOH bending frequency are suggested to occur [21,23].

The barcode (Figure 3c) shows shifts between EBC samples collected in both the baseline and infected stages in five of the 12 known WAMACS in the coordinates C1, C5, C7, C9, and C11 likely related to changes in the ratios of volatile and non-volatile compounds. In the normalized absorbance from EBC collected during the infected stage, NIR spectral peaks were right-shifted in C5 (1398–1418 nm), C7 (1432–1444 nm), and C11 (1482–1495 nm), more specifically to 1405, 1443, and 1490 nm, in comparison with initial peaks at 1398, 1443, and 1489 nm found in spectra of the baseline samples, suggesting a shift towards free water (C5), water dimers (C7), and water clusters with four hydrogen bonds (C11) [21,23]. At C1 (1336–1348 nm) and C9 (1458–1468 nm), NIR spectral peaks were left-shifted in samples from the infected stage at 1346 and 1458 nm in comparison to 1348 and 1489 nm during baseline, thus indicating a shift towards molecules structured in water asymmetrical stretching vibrations (ν_3_) and water clusters with two hydrogen bonds (S_2_) [21,23].

### 3.2. Discriminant Analysis Results

**Figure 4:**
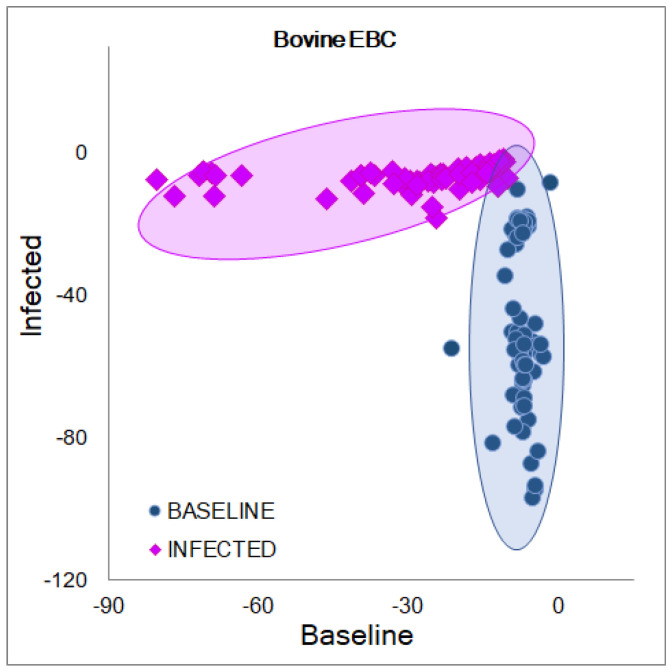
PCA-LDA plot for the calibration of Model 4 developed with the transformed absorbance (1300–1600 nm) from EBC collected before and after the BRSV challenge.

The results from the PCA-LDA models for classifying NIR-transformed absorbance (1300–1600 nm) of EBC collected from calves before and after the BRSV infection can be found in Table 1. On average, 7 ± 2 PCs explaining 99.6 ± 0.4% of the variation of the database were selected to perform the analyses. In this case, no significant differences (*p* < 0.05) were detected for the values of correctly predicted categories between the models (1–5) or between stages (baseline and infected) when applying the ANOVA or the Student’s t-tests. When evaluating the quality parameters from all the models simultaneously, significant differences (*p* < 0.05) were detected for the calibration values in comparison to the internal validation and the external validation. During the calibration, percentages of accuracy, sensitivity, and specificity of 97 ± 6, 98 ± 4, and 96 ± 9%, respectively, were achieved, indicating that 2 ± 4% of the transformed absorbance from samples of the infected stage were classified as false negatives, and 4 ± 9% of the transformed absorbance from samples of the baseline stage were classified as false positives. In the internal validation, the percentages 96 ± 8, 99 ± 3, and 93 ± 17% were obtained for the accuracy, sensitivity, and specificity, respectively. Hence, 1 ± 3% of the transformed absorbance from samples of the infected stage were classified as false negatives, and 7 ± 17% of the transformed absorbance from samples of the baseline stage were classified as false positives. Additionally, the external validation set containing the spectra from the calf excluded during the calibration was classified with percentages of 74 ± 21, 71 ± 27, and 76 ± 23%, for the accuracy, sensitivity, and specificity, correspondingly. Here, 29 ± 27% of the transformed absorbance were classified as false negatives, and 26 ± 23% were false positives to the viral infection. All the calibration PCA-LDA plots showed similar tendencies where two well-defined groups were found; in Figure 4, Model 4, omitting Calf 4, is shown as a representative of the five performed models. These results suggest that the biochemical differences found in the aquaphotomics evaluation due to changes in EBC composition during the infection can be discriminated using chemometrics-based MVA methods with quality parameters higher than the traditional clinical signs (VCD) and serological methods to detect this infection.

## 4. Discussion

The exhaled breath condensate (EBC) comprises >99.9% condensed water vapor and <0.1% aerosols [29,30]. While the condensing water absorbs water-soluble volatile compounds, respiratory droplets containing non-volatile molecules compose a minor fraction of the condensate [29,30]. The use of EBC as a biofluid to evaluate and detect respiratory diseases in small and large animals has gained special relevance in veterinary medicine [28,29]. The chemical composition of EBC from healthy and sick calves has been previously reported [28,29,30]. In healthy animals, hydrogen peroxide (H_2_O_2_), which is a volatile molecule in EBC, is well-known for its involvement in airway homeostasis [45,46]. During respiratory disease, H_2_O_2_ in EBC is considered a biomarker of inflammation and oxidative stress caused by the release of reactive oxygen species (ROS) and nitrogen species (RNS) from inflammatory leukocytes (neutrophils, eosinophils), monocytes (macrophages), and airway epithelial cells [30,47]. EBC from Holstein calves with bronchopneumonia (*n* = 15) also demonstrated higher levels of H_2_O_2_ in comparison to healthy neonatal calves (*n* = 15) [33]. Similarly, significant increases (*p* < 0.05) in H_2_O_2_ were detected in EBC collected from Holstein calves with mild (*n* = 20) and aggravated (*n* = 20) respiratory disease (*n* = 20) [32]. The increase in H_2_O_2_ causes bronchoconstriction and coughing when the mucous membranes of the trachea and bronchi become irritated as a result of their heightened sensitivity [32,48]. Here, the right shifts in the C5 and C7 WAMACS and WASPs from the aquaphotomics analysis are in alignment with increases in H_2_O_2_ found in infected individuals.

The eicosanoid leukotriene B_4_ (LTB_4_) is a pro-inflammatory mediator produced by neutrophils, eosinophils, macrophages, and epithelial cells that have been stimulated [28,49]. Leukotriene B_4_ (C_20_H_32_O_4_) is a known non-volatile component of bovine EBC and is considered a biomarker of inflammation during respiratory disease [28,29,30,49,50]. Increased levels of LTB_4_ in EBC were detected in two out of four calves infected with BRSV in comparison with baseline values [49], similar to calves with *Pasteurella multocida* serovar *D*, which also led to an increase in LTB_4_ after infection [49,50]. The observed changes in the aquaphotomics parameters, a left shift of C1 and C9, for inducing localized water structure would be consistent with increases in LTB_4_, and other lipid-based compounds known to increase in response to infection. Urea (CH_4_N_2_O) and ammonia (NH_3_) are other constituents of bovine EBC [26,51,52], and during homeostasis, the concentration of these compounds is related to food intake and ventilation [52]. However, the EBC from calves with induced bacterial pneumonia (*n* = 20) showed an increase in urea and ammonia, which was attributed to a change in the permeability of the lung-capillary barrier [52]. Other volatile organic compounds, such as acetaldehyde (C_2_H_4_O) and decanal (C_10_H_20_O), have also been reported in the EBC from steers (*n* = 3) diagnosed with BRDC by clinical signs (VCD), while heptane (C_7_H_16_), octanal (C_8_H_16_O), 2,3-butadione (C_6_H_10_), hexanoic acid (C_6_H_12_O_2_), and phenol (C_6_H_6_O) were associated with healthy animals (*n* = 3) [14]. More volatile compounds, as well as some chemical elements, have been detected at significant levels in bovine EBC using an electronic nose, finding potential biomarkers for animals with respiratory disease [31] that can be associated with the aquaphotomics profile found in the present study. The compounds 1,2-dimethylcyclohexylamine (C_8_H_17_N), aliphatic acids C_2_–C_5_, ammonia (NH_3_), cyclohexylamine (C_6_H_13_N), ethandal, methylamine (CH_3_NH_2_), methylglyoxal (C_3_H_4_O_2_), and pyridine-carbaldehyde (C_6_H_5_NO) were detected in calves with respiratory disease in comparison to healthy calves that only exhaled alkyl-, cyclic amines [31]. Chemical elements evaluated in the EBC from 18 sick Holstein calves with bronchopneumonia showed a significant decrease (*p* < 0.05) in Se and Zn, and an increase in Al, Co, Mn, Mo, Na, P, Pb, and Sn in comparison to the EBC from 12 healthy subjects [16].

Biofluid spectroscopy is a novel clinical research technology that provides a straightforward approach to gathering diagnostic information from easily obtained samples [19,53,54,55]. The NIR spectra of biofluids provide a plethora of information and may be viewed as a biochemical fingerprint of the sample state [56,57,58]. This is the first report on the use of NIRS to successfully evaluate and discriminate BRSV infection from EBC for disease diagnosis in animals. The decrease in the values for the external validation in the PCA-LDA are likely related to each individual calf’s immune response contributing to different biochemical changes for the classification of the samples. The chemical profile found in the aquaphotomics evaluation, and chemometric-based MVA analyses of NIR spectra are likely the result of the absorbance of the functional groups O-H, C-H, and N-H forming the molecules structuring the volatile and non-volatile compounds described for EBC from healthy and sick calves with respiratory disease.

## 5. Conclusions

NIR-aquaphotomics profiles were established for EBC collected from dairy calves challenged with BRSV based on the clinical signs following infection and the associated biochemical changes occurring with cell signaling and immune response activation. The aquaphotomics evaluation revealed changes in the organization of the aqueous phase of EBC acquired during the baseline and infected stages in the WAMACS C1, C5, C9, and C11 likely to be associated with both, the volatile and non-volatile compounds conforming this biofluid. Furthermore, the discrimination of the chemical profiles by PCA-LDA models distinguished EBC from pre-infected and infected calves with an accuracy, sensitivity, and specificity >93% in the calibration and validation. Here, the potential of NIRS in combination with aquaphotomics and chemometrics-based MVA to profile and discriminate this viral infection in unprocessed samples was demonstrated, providing evidence that the method could be used to develop an innovative, rapid, and non-invasive tool to support more effective diagnosis strategies for this problematic disease.

## Figures and Tables

**Table 1:** PCA-LDA for transformed absorbance (1300–1600 nm) classification and quality parameters for bovine EBC collected before and after the BRSV challenge.

Model	# Selected PCs	% Explained Variance	Category and Quality	% PCA-LDA Mahalanobis		
				Cal 80%	Val 20%	External Validation
1 (Calf 1 out)	8	99.8	baseline	64/64	16/16	7/20
			infected	64/64	16/16	9/20
			% Accuracy	100	100	40
			% Sensitivity	100	100	45
			% Specificity	100	100	35
2 (Calf 2 out)	9	99.9	baseline	64/64	16/16	16/20
			infected	64/64	16/16	20/20
			% Accuracy	100	100	90
			% Sensitivity	100	100	100
			% Specificity	100	100	80
3 (Calf 3 out)	7	99.6	baseline	64/64	16/16	18/20
			infected	64/64	15/16	10/20
			% Accuracy	100	97	70
			% Sensitivity	100	94	50
			% Specificity	100	100	90
4 (Calf 4 out)	5	98.9	baseline	54/64	10/16	17/20
			infected	59/64	16/16	20/20
			% Accuracy	86	81	93
			% Sensitivity	92	100	100
			% Specificity	80	63	85
5 (Calf 5 out)	8	99.9	baseline	63/64	16/16	18/20
			infected	64/64	16/16	12/20
			% Accuracy	99	100	75
			% Sensitivity	100	100	60
			% Specificity	98	100	90
Mean ± SD	7 ± 2	99.6 ± 0.4	% Accuracy	97 ± 6 (a)	96 ± 8 (a,b)	74 ± 21 (b)
			% Sensitivity	98 ± 4 (a)	99 ± 3 (a)	71 ± 27 (b)
			% Specificity	96 ± 9 (a)	93 ± 17 (a)	76 ± 23 (a)

Values with different letters were significantly different (*p* < 0.05) between the calibration (Cal 80%), the internal validation (Val 20%), and the external validation. No significant differences were detected in the prediction values between models after applying ANOVA and Tukey-Kramer HSD (honestly significant difference).

## Data Availability

Data are available upon request from Carrie K. Vance (ckv7@msstate.edu).

## Sample Availability

EBC Samples are available upon request from Carrie K. Vance (ckv7@msstate.edu).
